# Robotic assisted laparoscopic partial nephrectomy using contrast‐enhanced ultrasound scan to map renal blood flow

**DOI:** 10.1002/rcs.1738

**Published:** 2016-03-07

**Authors:** Ahmad Alenezi, Aamir Motiwala, Susannah Eves, Rob Gray, Asha Thomas, Isabelle Meiers, Haytham Sharif, Hanif Motiwala, Marc Laniado, Omer Karim

**Affiliations:** ^1^ Department of Urology, Frimley Health NHS Foundation TrustWexham Park HospitalSloughBerkshireSL2 4HLUK; ^2^ HCA Laboratories43a Wimpole StreetLondonW1G 8AQUK; ^3^ Department of PathologyWexham Park HospitalSloughBerkshireSL2 4HLUK

**Keywords:** robot assisted laparoscopic partial nephrectomy, selective ischaemia, global ischaemia, contrast enhanced ultrasounds scan (CEUS), microbubbles, SonoVue

## Abstract

**Objective:**

The paper describes novel real‐time ‘in situ mapping’ and ‘sequential occlusion angiography’ to facilitate selective ischaemia robotic partial nephrectomy (RPN) using intraoperative contrast enhanced ultrasound scan (CEUS).

**Materials and methods:**

Data were collected and assessed for 60 patients (61 tumours) between 2009 and 2013. 31 (50.8%) tumours underwent ‘Global Ischaemia’, 27 (44.3%) underwent ‘Selective Ischaemia’ and 3 (4.9%) were removed ‘Off Clamp Zero Ischaemia’. Demographics, operative variables, complications, renal pathology and outcomes were assessed.

**Results:**

Median PADUA score was 9 (range 7–10). The mean warm ischaemia time in selective ischaemia was less and statistically significant than in global ischaemia (17.1 and 21.4, respectively). Mean operative time was 163 min. Postoperative complications (n = 10) included three (5%) Clavien grade 3 or above. Malignancy was demonstrated in 47 (77%) with negative margin in 43 (91.5%) and positive margin in four (8.5%). Long‐term decrease in eGFR post selective ischaemia robotic partial nephrectomy was less compared with global ischaemia (four and eight, respectively) but not statistically significant.

**Conclusions:**

This technique is safe, feasible and cost‐effective with comparable perioperative outcomes. The technical aspects elucidate the role of intraoperative CEUS to facilitate and ascertain selective ischaemia. Further work is required to demonstrate long‐term oncological outcomes. © 2016 The Authors. *The International Journal of Medical Robotics and Computer Assisted Surgery* published by John Wiley & Sons, Ltd.

**Abbreviations and acronyms**
CEUS: contrast enhanced ultra sound
WIT: warm ischaemia time
PN: partial nephrectomy
RPN: robotic partial nephrectomy
LPN: laparoscopic partial nephrectomy
ICG: Indocyanine Green

## Introduction

The advantages of nephron‐sparing surgery over radical nephrectomy is well established with a pool of data providing strong evidence of oncological and survival equivalency 1, 2. After nephron‐sparing surgery, long‐term renal function relies on three fundamental factors: baseline renal function, volume of preserved nephrons and warm ischaemia time (WIT) 3, 4. WIT remains a subject of continuous debate. Review of the literature proposed limiting warm ischaemia to <20 mins if feasible and adopting it as an acceptable threshold to prognosticate post‐operative renal function 5, 6. Considering the superior dexterity and vision, shorter learning curve and comparable outcomes, robotic surgery has currently established itself as a valuable modality in renal surgery 7. Various robotic surgical techniques have been developed to further reduce renal ischaemia and minimize perioperative complications while maintaining sound oncological outcomes 8, 9, 10, 11, 12, 13.

At our institution, we have described a novel technique to road map blood flow to the kidney by incorporating real‐time intraoperative contrast‐enhanced ultrasound scan (CEUS) 10. In essence, the efforts have focused on refining the technique to facilitate and ascertain global or selective ischaemia prior to transperitoneal robotic partial nephrectomy (RPN) and to potentially reduce WIT. In this article we describe two further developments of the technique. We also present and evaluate our initial series and perioperative outcomes.

## Materials and methods

### Study population

Transperitoneal RPN was performed in 60 patients (61 tumours) between 2009 and 2013. Data were prospectively collected and retrospectively assessed. Patient demographics are demonstrated in Table 1. A descriptive statistical analysis was performed using the GraphPad Prism software program.

**Table 1:** Demographics of study cohort

**Age**	
Mean (SD)	56 (15.3)
Median (IQR)	58 (44‐66)
**Male patients, no. (%)**	50 (83.3)
**ASA score**	
Mean (SD)	1.67 (0.63)
Median (IQR)	2 (1‐2)
**Co‐morbidities, no (%)**	24 (40)
Hypertension	13 (21.7)
Ischaemic heart disease	3 (5.0)
Hyperchlosterolaemia	7 (11.7)
Diabetes mellitus	5 (8.4)
Asthma, COPD	4 (6.7)
ConT disease (sarcoidosis, PMR)	2 (3.3)
Other cancer (rectal , prostate)	5 (8.4)
Not recorded	6 (10)
**Presentation, no (%)**	
Incidental (LUTS, health screen, cardiac imaging, research volunteer, testicular pain, weight loss)	34 (56.7)
Haematuria	8 (13.3)
Urinary tract infection	5 (8.3)
Loin pain	4 (6.7)
Surveillance scan (other cancer)	4 (6.7)
Not recorded	5 (8.3)

SD = standard deviation; IQR = Inter Quartile Range; ASA = American Society of Anesthesiology; COPD = chronic obstructive pulmonary disease; ConT = connective tissue; PMR = polymyalgia rheumatica; LUTS = lower urinary tract symptoms

### Preoperative plan

**Figure 1:**
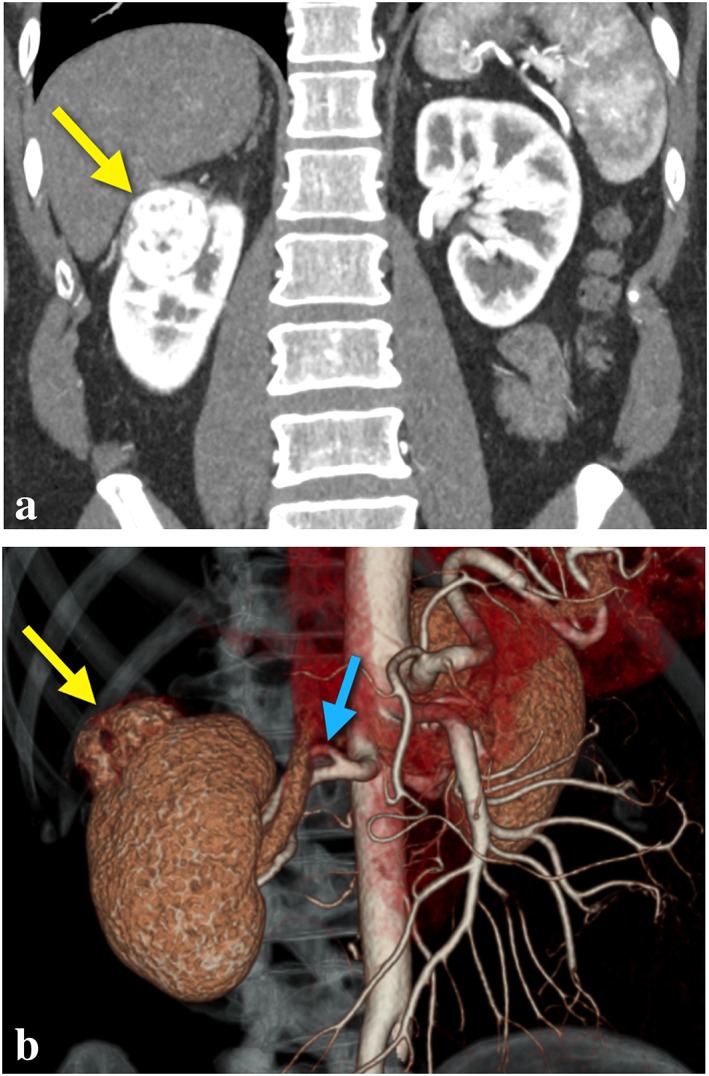
(a) Coronal CT scan demonstrating a predominantly endophytic right upper pole renal tumour (yellow arrow). (b) Same renal tumour (yellow arrow) with 3D reconstruction of CT renal arteriogram identifying upper pole branch of renal artery (blue arrow) suitable for selective occlusion RPN

Medical history, examination and investigations were included. A three‐dimensional (3D) abdominal computed tomography (CT) urogram with 2–3 mm cuts was performed. When visualization of the vasculature was inadequate, a 3D CT renal angiogram (CTA) was requested to delineate the anatomy of the renal artery branches, venous tributaries and their relationship to the tumour and surrounding structures. A PADUA score was created as illustrated in Table 2. The decision for RPN selective versus global ischaemia was determined based on tumour location and the arterial ‘road map’ radiological findings (Figure 1). Serum creatinine was documented pre‐operatively, < 1 week early post‐operatively and > 1 month ‐ < 18 month late post‐operatively. Estimated glomerular filtration rate (eGFR) was calculated using the modification of diet in renal disease (MDRD) formula. Estimated GFR was recorded pre‐operatively, < 1 week early post‐operatively and > 1 month ‐ < 18 month late post‐operatively.

**Table 2:** Characteristics of tumours

**Tumour, no.**		61	
Solitary kidney, no. (%)		3 (4.9)	
CT scan tumour size, mm			
Mean (SD)		36 (15.1)	
Median (IQR)		34 (24 ‐ 45)	
Tumour on right side, no. (%)		35 (57.4)	
Tumour cystic or solid, cystic no. (%)		7 (11.5)	
Tumour upper, middle, lower pole, no. (%)		18, 20, 23 (29, 33, 38)	
Number of renal arteries 1, 2 or 3, no, (%)		46, 14, 1 (75, 23, 2)	
PADUA score			
Mean (SD)		8.8 (1.9)	
Median (IQR)		9 (7 ‐ 10)	
Intraoperative ultrasound used, no (%)		54 (88.5)	
CEUS used, no (%)		38 (62.3)	
TNM Stage by Ischaemia	Global	Selective	Off‐clamp
pT1a	20	17	1
pT1b	2	2	0
pT2	1	0	0
pT3a	3	1	0
Benign	5	7	2
PADUA Classification Variables			
Longitudinal (polar) location	*Superior/Inferior (1)*	*Middle (2)*	
	9 (14.8%)	52 (85.2%)	
Exophytic rate	*≥50% (1)*	*≤50% (2)*	*Endophytic (3)*
	28 (45.9%)	28 (45.9%)	5 (8.2%)
Renal Rim	*Lateral (1)*	*Medial (2)*	
	46 (75.4%)	15 (24.6%)	
Renal Sinus	*Not Involved (1)*	*Involved (2)*	
	38 (62.3%)	23 (37.7%)	
Urinary Collecting System	*Not Involved (1)*	*Dislocated/Infiltrated (2)*	
	41 (67.2%)	20 (32.8%)	
Tumour size (cm)	*≤4 (1)*	*4.1‐7 (2)*	*>7 (3)*
	42 (68.9%)	16 (26.2%)	3 (4.9%)
Anterior/Posterior	*Anterior*	*Posterior*	
	44 (72.1%)	17 (27.9%)	
Total Score	Low (6‐7)	Medium (8‐9)	High (≥10)
	19 (31.1%)	22 (36.1%)	20 (32.8%)

CT = computed tomography; SD = standard deviation; IQR = Inter Quartile Range;

### Operative approach

A robotic four‐arm transperitoneal approach was performed in all patients. Additional 12 mm and 5 mm assistant ports were used. An insufflation pressure of 10 mmHg was used except at the time of tumour excision and renorrhaphy, when the pressure was temporarily increased to 20 mm Hg to facilitate hemostasis.

### Surgical technique

After mobilizing the bowel, hilar dissection was performed to separate the renal artery branches. The branches were individually colour‐coded with vessel loops. Intraoperative ultrasound scan with the ProART robotic drop‐in probe was used to identify the tumour. Gerota's fascia was entered some distance away from the tumour, and dissection just above the surface of the kidney. Fat and Gerota's fascia were preserved on the tumour for accurate pathologic staging and to aid in retraction. The ultrasound drop‐in probe was reinserted through the assistant port. Scanning of the tumour and intra‐parenchymal depth were assessed.

In certain kidneys with either an upper‐ or lower‐pole tumour, the main arterial supply to the region surrounding the tumour was relatively obvious with a separate polar branch. In this situation, the branch was selectively clamped with bulldog clamps. With the arterial clamp *in situ*, 1 mL of SonoVue® (Bracco International, Netherlands), was injected intravenously. The scan was switched to perform in the SonoVue contrast mode (low mechanical index (MI)) to avoid destruction of the microbubbles.

Circulation or lack of circulation within the segment containing the tumour images was monitored at the console in TilePro mode. Excision of the tumour was performed using cold cut scissors and ProGrasp forceps. A 2‐0 V‐Loc suture with an anchoring Hem‐o‐lok clip in the loop of the suture was used to oversaw renal parenchymal vessels in the renal bed. The defect in the renal cortex was closed with No. 1 on a CT1 needle using sliding‐clip renorrhaphy 14.

### Assessment of renal perfusion using contrast enhanced ultrasound scan (CEUS)

Prior to injection of SonoVue, in CEUS mode, the kidney appears characteristically dark and devoid of the echogenic SonoVue microbubbles. When SonoVue is injected with the artery selectively clamped, subsequently, a clear zone of demarcation or watershed, developed between the perfused and non‐perfused segments.

**Figure 2:**
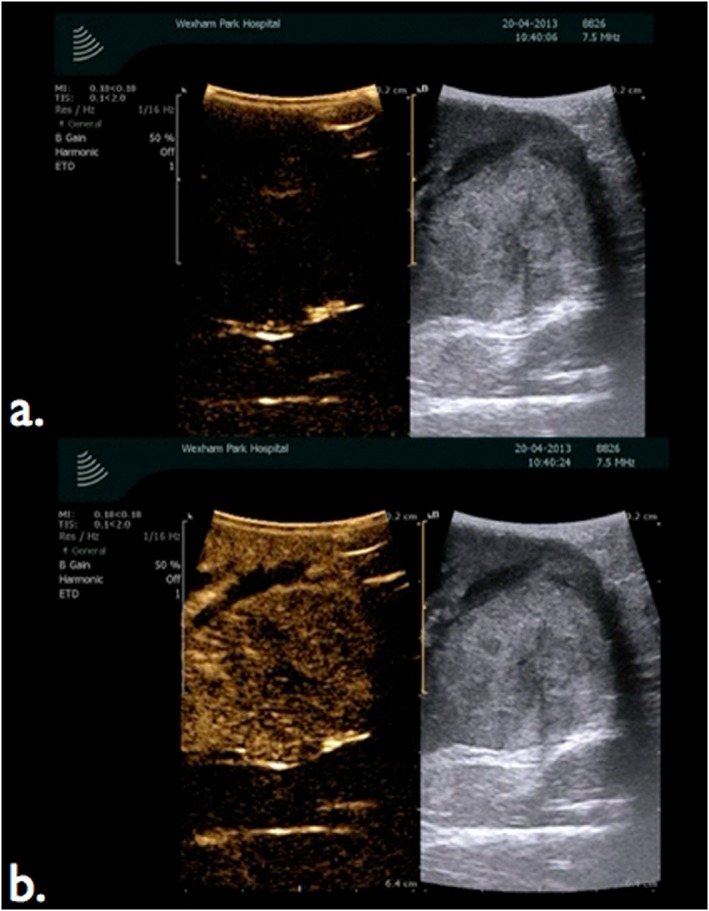
*In situ* mapping of renal blood flow using B mode ultrasound image of renal tumour (right) with contrast enhanced ultrasound mode (left). (a) Prior to injection of SonoVue or with selective occlusion of a branch of the renal artery supplying the region of the tumour or (b) increase in echogenicity of tumour and renal parenchyma 20 s after intravenous injection of SonoVue demonstrating blood flow when the branch of the renal artery supplying the region of the tumour is not clamped

An ultrasound scan was performed in contrast mode (probe frequency 4 Hz with low MI). The low MI prevents disruption of the SonoVue microbubbles. Within 20 s of an intravenous injection, the echogenic contrast was seen to perfuse the kidney, without any devascularization (Figure 2).

### In situ mapping of renal blood flow using intra‐operative CEUS

Based on preoperative CTA, the ‘key or index’ artery supplying the concerned region of the tumour is dissected and isolated in a colored vessel loop. The artery is temporarily occluded with a robotic (Scanlan, Minnesota, USA) or laparoscopic (Aesculap, Melsungen, Germany) bull‐dog clamp. Renal perfusion is ascertained as illustrated above. In the learning phase, if there is doubt further injection of SonoVue may be given multiple times to ascertain devascularization of the region containing the tumour by clamping and unclamping the isolated branch (Figure 2). If devascularization of the tumour and surrounding kidney was inadequate following intraoperative CEUS assessment, then the RPN is performed using global ischaemia, by clamping the main renal artery.

### Sequential selective occlusion angiography using CEUS and destruction of microbubbles and specimen excision

If there is persistent flow around the tumour we instigate ‘sequential selective occlusion angiography’ whereby, after each flow assessment, the bull‐dog clamp is removed and the SonoVue microbubbles are destroyed. This is achieved by temporarily increasing the frequency of the ultrasound from 4.0 to 8.5 MHz. This has the effect of increasing the mechanical index (MI) of ultrasound waves from 0.18 to 1.9 and disrupting the microbubbles. After about two min scanning in high frequency mode (8.5 Hz), assessment of blood flow using the above CEUS process can be repeated again in what is effectively a ‘fresh’ or contrast‐free field of renal parenchyma. The bull‐dog clamp is reapplied on a more proximal or distal (tertiary) division of an anterior or posterior renal artery branch (sequential selective occlusion) in order to achieve a more discreet area of devascularization of the tumour.

**Figure 3:**
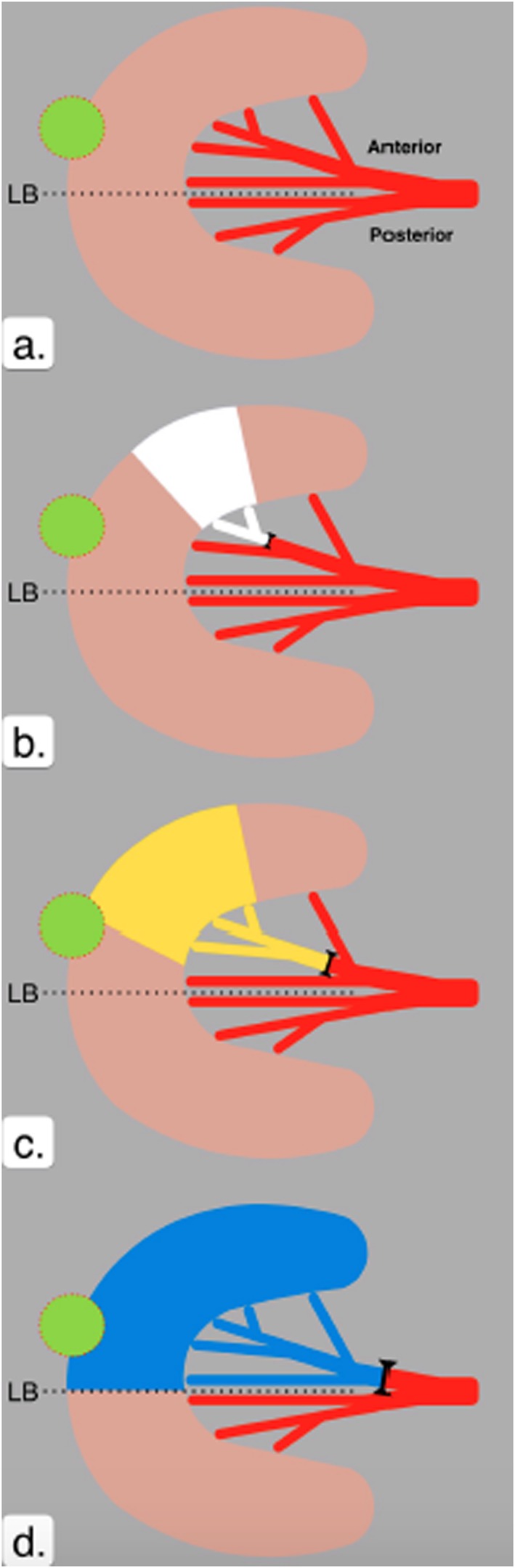
A schematic axial diagram of a kidney to demonstrate the principle of sequential selective occlusion angiography using CEUS (contrast enhanced ultrasound scan). (a) The main renal artery divides into anterior and posterior divisions. The small renal tumour is represented as the green circle circumscribed with dotted red line. The avascular plane or line of Brödel is represented by the dotted line LB. (b) and (c) show the area of ischaemia induced by sequential occlusion of a distal (white) then more proximal division (yellow) of the renal vessel. (d) With occlusion of the anterior division of the renal artery (blue), the area of the tumour is made ischaemic and the segment of the kidney, lying posterior to the line of Brödel is still perfused

**Figure 4:**
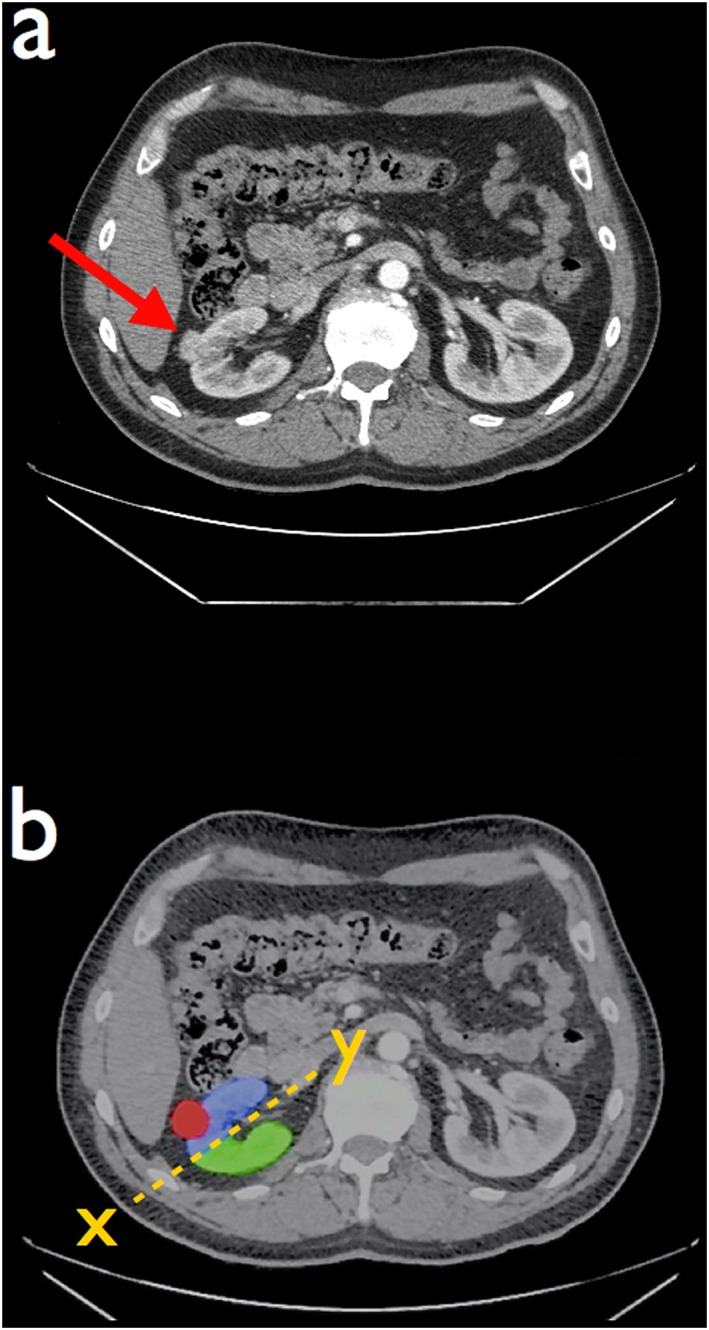
(a) Axial CT scan demonstrating a small tumour on the anterior aspect of the right kidney (red arrow). (b) Schematic colouring of CT scan to demonstrate the position of a tumour (red circle) and renal parenchyma anterior (blue) or posterior (green) to the avascular plane of Brödel denoted by the dotted yellow line X–Y. The tumour is located in the area served by the anterior division (blue), the branches of which supply the region of the tumour

**Figure 5:**
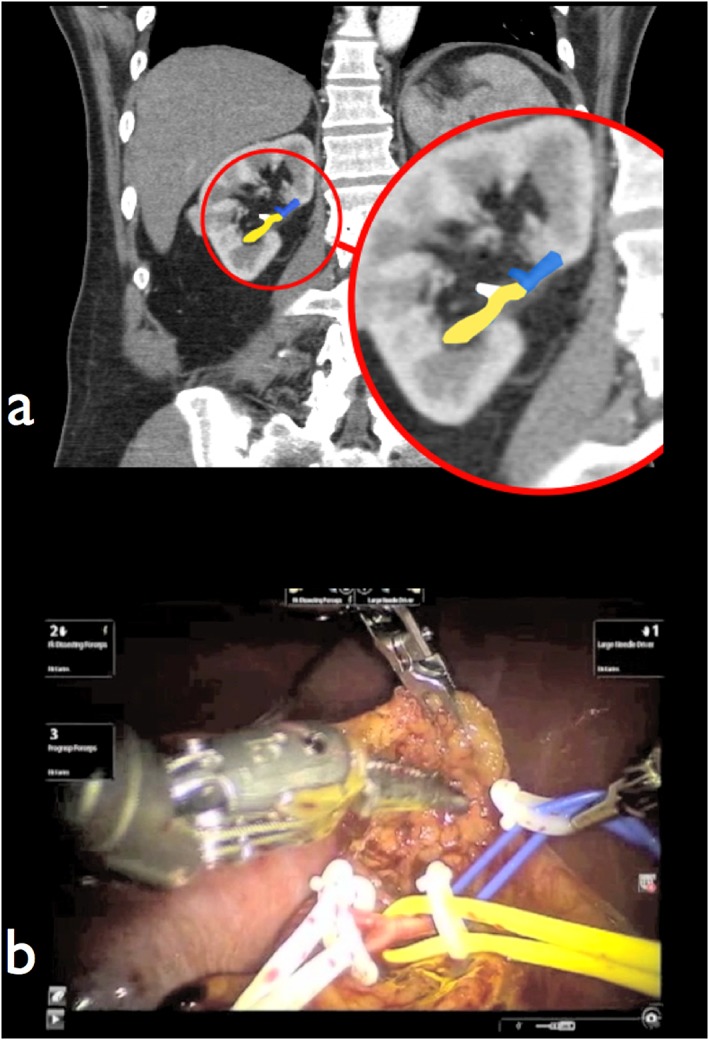
(a) Coronal CT scan of right kidney demonstrating colour‐coded primary or anterior (blue), secondary (yellow) and tertiary (white) divisions of the right renal artery. (b) Equivalent intraoperative image with the same colour coded plastic slings round the corresponding vessels

**Figure 6:**
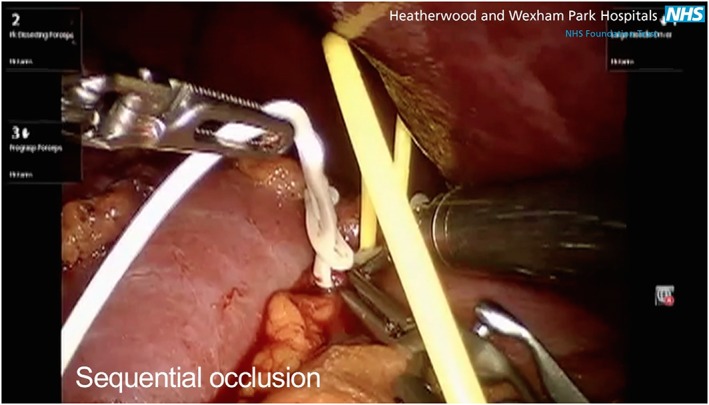
Sequential selective occlusion angiography of the distal tertiary division (white vessel loop)

**Figure 7:**
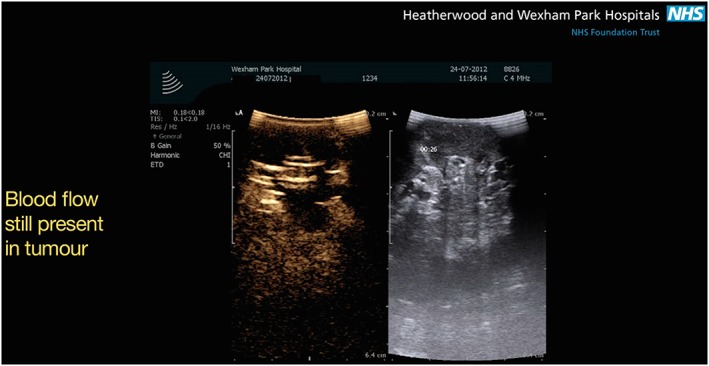
After injection of the microbubble the area of the kidney containing the tumour remained perfused indicating that there was inadequate devascularization

**Figure 8:**
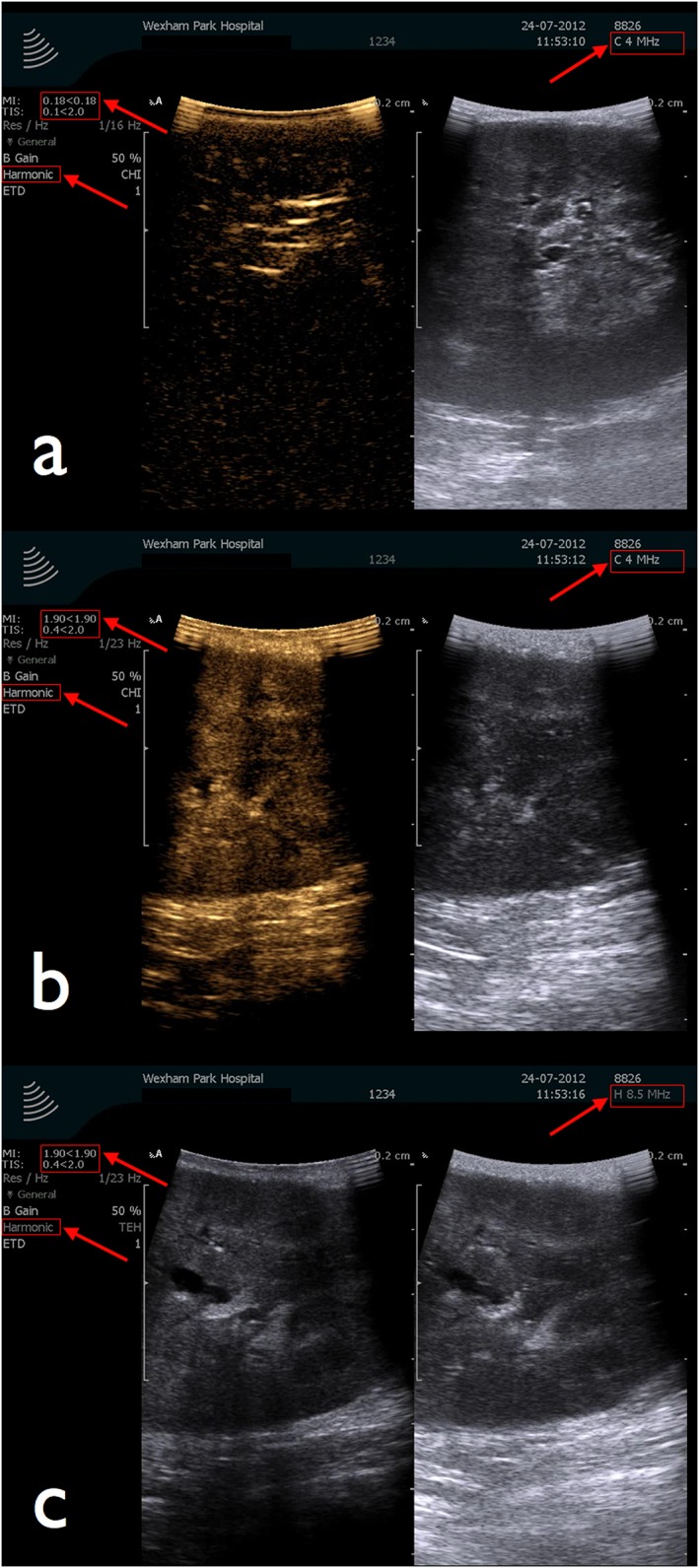
Ultrasound sequences demonstrating steps used to lyse SonoVue® microbubbles on each occasion, prior to repeating an injection of SonoVue®. B‐mode (on right) and contrast enhanced (on left) ultrasound images of the kidney. (a) Post‐injection of IV SonoVue® in contrast enhanced ultrasound mode (probe frequency is 4 Hz) image shows echogenicity of perfused renal parenchyma. (b) Probe frequency in the process of being increased from 4 to 8.5 Hz. This process increases the mechanical index (MI) of the ultrasound waves to intentionally disrupt the SonoVue® microbubbles. The MI is seen to change from 0.18 (6(a)) to 1.9 (6(b) and 6(c)). (c) With the higher frequency (8.5 Hz) the contrast ultrasound mode is non‐operational and both left and right images from the split screen show a B‐mode ultrasound image

**Figure 9:**
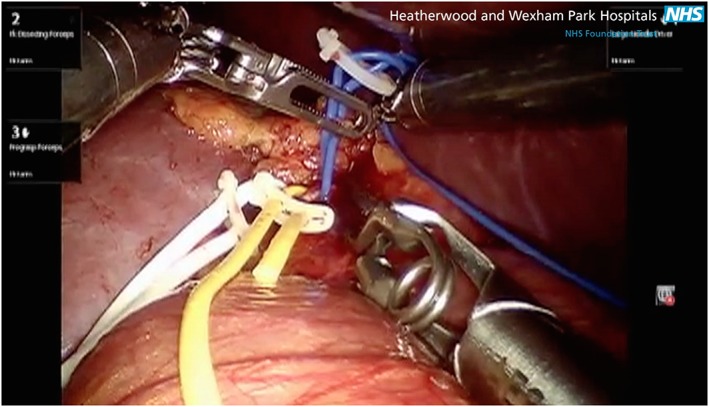
Sequential selective occlusion of the more proximal branch (secondary or primary division) and repeat injection of SonoVue and scanning until adequate devascularization of the area of the kidney containing the tumour is achieved to allow tumour excision

Figures 3, 4, 5, 6, 7, 8, 9 are schematic, radiological and intraoperative images that illustrate the details of the steps. In this example the right renal artery divides into an anterior and posterior division, which for clarity are colour coded blue and green respectively. Figures 3 and 4 show that the tumour is located in the area served by the anterior division (blue), the branches of which supply the region of the tumour. Figure 5 shows colour coded vessel loops, which correspond to the branches of the anterior division of the renal artery. The blue vessel loop is on the anterior division (primary division). The yellow vessel loop is placed around a secondary division and the white vessel loop is around a tertiary division. With all vessel loops in place, the area of the kidney supplied by each division branch can then be assessed using CEUS microbubble material. Figure 6 shows sequential selective occlusion of the distal tertiary division (white vessel loop). After injection of the microbubble the area of the kidney containing the tumour remained perfused (Figure 7), indicating that there was inadequate devascularization. Lysis of the SonoVue microbubble was then performed as shown in Figure 8, in order to repeat the process this time by sequential selective occlusion of the more proximal branch (secondary or primary division) and repeat injection of SonoVue and scanning until adequate devascularization of the area of the kidney containing the tumour is achieved to allow tumour excision, Figure 9. If the renal parenchyma exhibits significant arterial bleeding despite selective ischaemia, a clamp across the main renal artery can be placed and the operation is converted to a global ischaemia.

## Results

A total of 60 patients (61 tumours) were included in the analysis (Table 1). RPN was completed with Ischaemia time ‘zero’ (off clamp) in three, hilar clamping global ischaemia in 31 and without hilar clamping and selective in 27. Intra‐operative ultrasound scan was used in 54 (85.5%) of the patients and of those, intraoperative CEUS was used in 38 (62.3 %). Selective ischaemia CEUS with ‘in situ mapping’ and ‘occlusion angiography’ was used in 22 tumours and global ischaemia was used in 16. In the global ischaemia group six patients started with selective ischaemia approach then progressed to global ischaemia (Table 3).

**Table 3:** Cohort study distribution of intraoperative imaging

**Warm Ischemia**	**Global**	**Selective**	**Off‐clamp**	**Total**	**P value selective vs. global**
**I*maging Type***
**US only**	9 (81)	4 (96)	3 (100)	16	
**CEUS**	16 (52)	22 (81)	0 (0)	38	**0.01**
**No Imaging**	6 (19)	1 (4)	0 (0)	7	

SD = standard deviation; IQR = Inter Quartile Range

**Figure 10:**
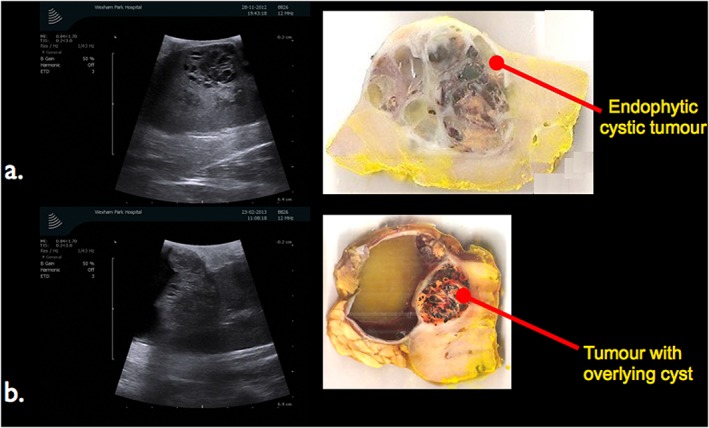
Intra‐operative ultrasound scan imaging of kidney to demonstrate location and nature of an endophytic cystic tumour and a tumour with large overlying cyst. Ultrasound imaging ensures tumour is excised with a small margin of healthy renal tissue

The mean tumour size was 3.6 cm (SD: 15.1). The median PADUA score was 9 (range 7–10) (Table 2). Seven (11.5%) of the tumours were either cystic or contained cysts (Figure 10).

The perioperative outcomes are summarized in Table 4. The mean tumour size was smaller in the selective ischaemia group at 31 mm (SD: 10.9) compared with the global ischaemia group at 43 mm (SD: 17.6) (*P* = 0.01). The mean WIT was 18.4 min for all RPN. However, the mean WIT in the selective ischaemia group was less and shorter in duration and statistically significant than in the global ischaemia group (17.1 and 21.4, respectively).

**Table 4:** Perioperative outcomes

	**Overall**	**Global Ischaemia**	**Selective Ischaemia**	**Off clamp**	**p‐value Selective v Global**
**Patients (total = 60) with tumours, no. (%)**	61 (100)	31 (50.8)	27 (44.3)	3 (4.9)	
**Operative time, mins**					
Mean (SD)	163 (46.4)	176 (44.1)	154 (47.0)	126 (24.8)	0.10
Median (IQR)	153 (129‐196)	172 (138 – 206)	133 (118 ‐ 194)	126 (108 – 143)	
**CT scan size of tumour, mm**					
Mean (SD)	36 (15.1)	41 (16.6)	31 (10.9)	24 (18.2)	**0.01**
Median (IQR)	34 (24 ‐ 45)	37 (31 – 48)	29 (23 – 42)	31 (3 – 37)	
**PADUA score**					
Mean (SD)	8.8 (1.9)	8.9 (2.1)	8.9 (1.6)	6.7 (0.6)	0.97
Median (IQR)	9 (7 ‐ 10)	9 (7 – 11)	9 (8 – 10)	7 (6 – 7)	
**Warm ischaemia time, min**					
Mean (SD)	18.4 (6.7)	21.4 (5.4)	17.1 (3.9)	0	**<0.01**
Median (IQR)	19.0 (15.0 ‐ 22.0)	20.0 (18.0 – 24.5)	16.4 (14.7 – 19.7)	0	
**Estimated blood loss, ml**					
Mean (SD)	280 (262)	298 (250)	244 (279)	400 (265)	0.45
Median (IQR)	200 (100 ‐ 375)	200 (100 – 400)	175 (100 – 300)	500 (100 – 600)	
**Pre Op Creatinine**					
Mean (SD)	87 (18.4)	87 (20.4)	85 (15.2)	97 (23.8)	0.69
Median (IQR)	87 (75 ‐ 99)	87 (73 – 99)	83 (76 – 98)	101 (71 – 118)	
**Early Post Op Creatinine mmol/L (<1 week)**					
Mean (SD)	112 (42.1)	114 (36.0)	104 (27.0)	194 (154.2)	0.28
Median (IQR)	106 (89 ‐ 125)	106 (92 – 122)	106 (82 – 108)	194 (85 – 303)	
**Late Post Op Creatinine mmol/L (>1 month, <18 months)**					
Mean (SD)	95 (24.6)	97 (26.7)	90 (17.5)	113 (46.2)	0.24
Median (IQR)	97 (79 ‐ 104)	97 (83 – 107)	94 (77 – 103)	101 (74 – 164)	
**Pre Op eGFR ml/min 1.73 m2 (<1 week)**					
Mean (SD)	85 (21.5)	86 (25.6)	85 (15.0)	78 (28.0)	0.81
Median (IQR)	82 (72 ‐ 97)	81 (69 – 100)	85 (74 – 93)	69 (55 – 109)	
**Early Post Op eGFR ml/min 1.73 m2 (<1 week)**					
Mean (SD)	68 (21.7)	65 (19.8)	72 (21.6)	54 (50.2)	0.33
Median (IQR)	65 (53 ‐ 82)	65 (53 – 80)	70 (53 – 84)	54 (18 – 89)	
**Late Post Op eGFR ml/min 1.73 m2 (>1 month, <18 months)**					
Mean (SD)	78 (24.1)	77 (27.6)	80 (18.8)	70 (33.5)	0.68
Median (IQR)	72 (68 ‐ 90)	73 (66 – 87)	72 (69 – 93)	69 (37 – 104)	
**Long term decrease in eGFR ml/min 1.73 m2 (>1 month, <18 months)**					
Mean (SD)	6 (12.1)	8 (11.8)	4 (12.7)	12 (9.2)	0.32
Median (IQR)	5 (‐2 ‐ 14)	5 (‐1 – 14)	5 (‐3 – 12)	12 (5 – 18)	

The mean operative console time was 163 min for all RPN. The mean blood loss was 298 mL for global ischaemia but less (244 mL) in the selective ischaemia and was not statistically significant. No patient had intra‐operative transfusion or complication. Post‐operative complications (n = 10) included three (5%) Clavien grade 3 or above, one of which was a death in an elderly patient from a myocardial infarction. There were no conversions to open or laparoscopic surgery and the mean length of hospital stay was 3.6 days (STD: 2.04) (Table 5).

**Table 5:** Intra‐ and post‐operative complications

**Complications**	
Intra‐operative complication, no (%)	2 (3.34)
Organ injury (spleen tear)	1 (1.67)
Dropped instrument (vascular clamp)	1 (1.67)
Post‐operative complications, no. (%)	10 (16.7)
Grade I Ileus (1)	1 (1.67)
Grade II Transfusion (4), Pneumonia (2)	6 (10)
Grade III Urinary leak (1), Retained drain (1)	2 (3.33)
Grade IV	
Grade V Death from myocardial infarction (1)	1 (1.67)

Tumour histopathology is detailed in Table 6. Malignancy was demonstrated in 47 (77%) with negative margin in 43 (91.5%) and positive margin in four (8.5%). The mean pathology tumour size was 32 mm (SD: 14.5) with renal cell carcinoma clear cell histology representing the most frequent diagnosis of malignancy (85.1% of cases). Mean pre‐ and post‐operative serum creatinine for global ischaemia group was 87 mmol L^‐1^ and 97 mmol L^‐1^, respectively. Mean pre‐ and post‐operative serum creatinine for selective ischaemia group was 85 mmol L^‐1^ and 90 mmol L^‐1^, respectively. The long‐term decrease in estimated glomerular filtration rate (eGFR) post selective ischaemia RPN was 4 mL min^‐1^ per 1.73 m^2^ (SD: 12.7) and the global ischaemia RPN 8 mL min^‐1^ per 1.73 m^2^ (SD: 12.8). The decrease in eGFR was less in the selective ischaemia group compared with the global ischaemia group (four and eight, respectively) but not statistically significant (Table 4).

**Table 6:** Pathology

**All Patients**	
Malignant, no (%)	47 (77)
Benign, no (%)	14 (23)
Pathology tumour size, mm	
Mean (SD)	32 (14.5)
Median (IQR)	30 (22 ‐ 37.5)
Positive surgical margins, no (%)	4 (6.6)
**Malignant**	
RCC, clear cell	40 (85.1)
RCC, papillary	5 (10.6)
RCC, chromophobe	2 (4.3)
RCC pathologic stage	
pT1a	38 (80.9)
pT1b	4 (8.5)
pT2	1 (2.1)
pT3a	4 (8.5)
**Benign patients**	
Oncocytoma	9 (64.3)
Benign cyst	3 (21.4)
Angiomyolipoma	2 (14.3)

## Discussion

All contemporary PN techniques including open, laparoscopic and robotic, typically involved hilar clamping in order to achieve the desired bloodless operative field 15. While many research studies proposed that a limited warm ischaemia of <20–30 min may be transient and spontaneously reversible, others have debated that this transient ischaemia can be detrimental particularly in patients with pre‐existing comorbidities or renal impairment. Zero ischaemia technique was introduced to eliminate ischaemia to the functional renal tissue. The researchers employed a selective branch microdissection of dedicated tertiary or quaternary branch coupled with calibrated and timed intraoperative reduction of blood pressure 16. Another group described 7‐year experience with zero‐ischaemia laparoscopic partial nephrectomy (LPN) after super selective transarterial tumour embolization (STE). The procedure produced significant functional outcomes with less complications and better oncologic results 17.

In our experience, intraoperative CEUS offers superior imaging compared with other intraoperative modalities. CEUS performed with a robotic ultrasound probe has a simple and easy learning curve. Tumours generally have good vascularity, a feature which can enhance the quality of the signals detected with CEUS when compared with other ultrasound modalities such as power Doppler. When combining selective occlusion angiography with CEUS, CEUS was more useful in assessing the regions of ischaemia and perfusion.

The contrast microbubble agent used in the CEUS procedure is non‐allergenic and does not interfere with renal function, as it is not excreted by the kidneys. It is, therefore, not contra‐indicated in patients with impaired renal function. The kidney and the pelvicalyceal system have no role in the accumulation and excretion of the contrast agents. Furthermore, in conditions of reduced renal blood perfusion, ischaemia, and diabetic nephropathy; the short duration of uptake of this contrast agent can safely be overcome by administering the agent as multiple injections 18. In our study, we have safely re‐injected the SonoVue contrast agent at least twice or three times in all patients without any adverse reported outcomes.

In addition, CEUS have a distinct advantage over ICG in that, repeat doses of contrast material may be given within short periods or multiple times without adverse implications (ICG takes 20 min to wash away). Furthermore, with CEUS there is no risk of movement artifact as with Doppler ultrasound which can give a false impression of blood flow even in devascularized tissue 12.

FireFly fluorescence with ICG necessitates a specialized camera and equipment, which makes it very costly. Recently published literature demonstrated intraoperative CEUS to be more cost‐effective and economically feasible compared with ICG 18, 19. Sodium iodide is a constituent of ICG that potentially carries a risk of anaphylactic shock. Hence, it is imperative to exclude any previous history of allergic reactions. Safety research work on the microbubble SonoVue in more than 20 000 patients showed a potential serious event in 0.0086% 20.

As for tumour complexity, there was no difference in the PADUA score between the selective and global ischaemia groups. In our series, intraoperative CEUS demonstrated a shorter WIT in the selective ischaemia group and was statistically significant, likely due to smaller tumour size in the selective ischaemia compared with the global ischaemia group. Of note, our intraoperative blood loss in the selective ischaemia was similar to previously reported unclamping series 21; no patient in this series required transfusion. Also our series attained similar complication rate to other contemporary larger series 22.

In this study, our main objectives were to describe the technique of CEUS and the scope was to assess its practical application among selective versus global ischaemia RPN groups. Hence, a direct comparison between CEUS versus US alone was not performed. Moreover, the number of patients who underwent RPN using US alone in this series was also too small to allow a sub‐analysis comparison. Further studies to elucidate a direct comparison between CEUS and other existing intraoperative imaging modalities are warranted in the future.

We were keen to determine functional outcome benefits in patients undergoing selective versus global ischaemia. Paradoxically, our preliminary small series of patients did not demonstrate other measurable benefits of selective ischaemia (Table 4).

Several reasons could justify the findings. In this series we used eGFR; in a recent analysis Wiener *et al*. revealed that age, BMI > 30, estimated blood loss >200 mL, CCI > 5, and WIT were associated with greater declines in eGFR. Using multivariate analysis, only age was significantly associated with a decline in eGFR 23. Hence, relying solely on eGFR is a crude measure in assessing long‐term renal function.

The technique of sequential occlusion might be criticized for repeatedly clamping branches of the renal artery. Theoretically, there could be damage to the endothelium each time the clamp is re‐applied 24. The robotic bull‐dog clamps are however, designed specifically for atraumatic application. In fact, atheromatous plaques, which could increase the risk of mechanical trauma, are much less frequent beyond the ostium of the main renal artery. In addition, the influence of sequential intermittent versus continuous clamping of the renal pedicles on functional outcomes has neither shown benefit nor harm to renal function in animal studies 25, 26. Similarly published collaborative review has revealed that, to date, no clinical studies have demonstrated improvement in WIT or functional outcome after modifications of any clamping technique 27.

With experience, we successfully managed to reduce the operative time to around 12 minutes by performing the steps in the following cycle: applying the bull‐dog clamp, injecting the SonoVue, scanning the kidney, removing the bull‐dog clamp, rescanning in high resolution mode, and re‐injecting Sonovue. Hence the additional console time may be justified during RPN, especially in patients with a solitary or ‘precious’ kidney and in co‐morbid patients prone to high risk of ischemic renal damage.

**Figure 11:**
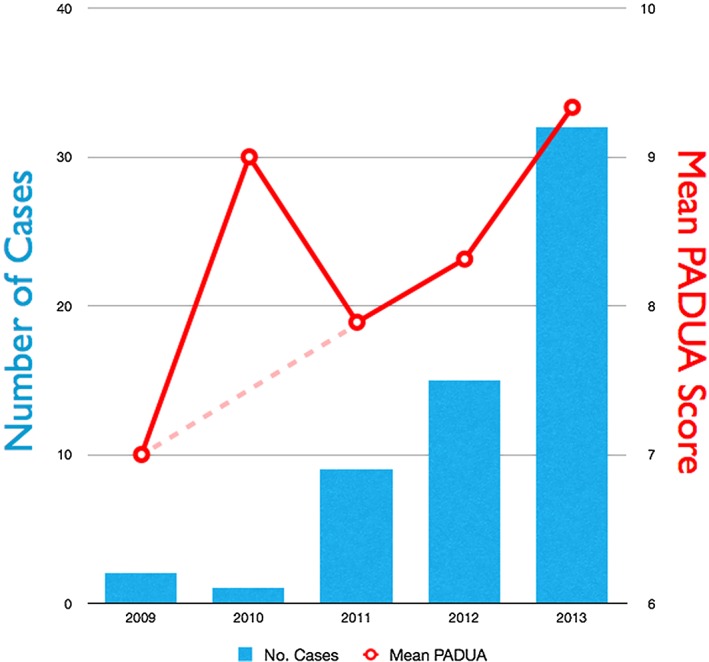
Graph to show the annual number of RALPN performed between 2009 and 2013 and the corresponding mean PADUA score of the renal tumours excised each year

**Figure 12:**
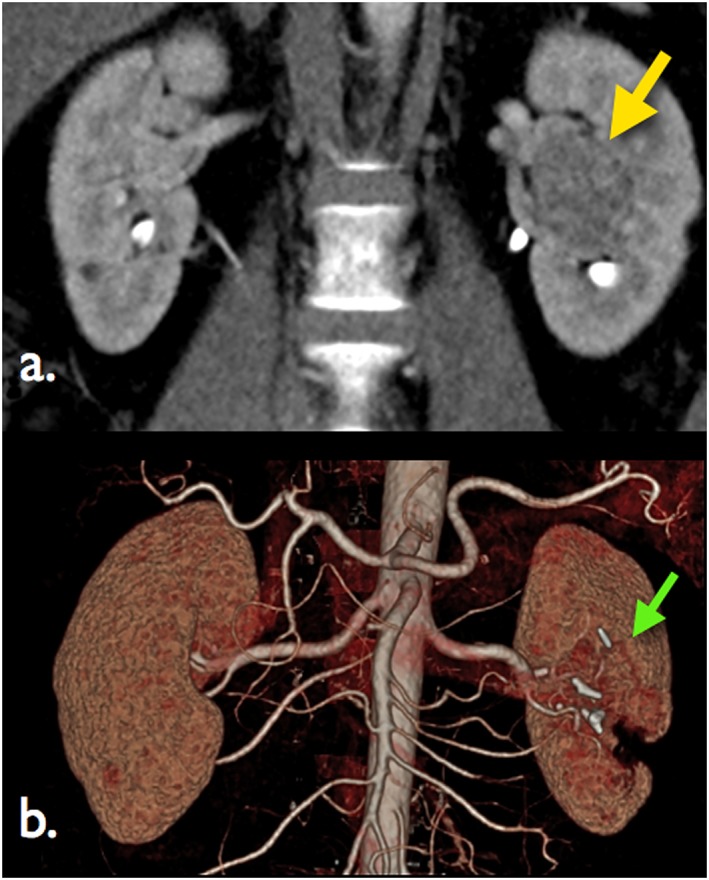
(a) Saggital section of complex hilar tumour (yellow arrow). (b) Post‐operative 3D reconstruction computed tomography scan to show scar defect in kidney (green arrow) post‐RALPN

Despite our encouraging results, an issue that needs emphasizing is the surgical margin. Negative surgical margin (NSM) was seen in 43 (91.5%) and positive surgical margin (PSM) in four (8.5%). The distance from tumour edge to the surgical margin was 10 mm on average in patients with positive margin and 4 mm on average in patients with negative margin. The rate of this PSM was divided evenly between both selective and global ischaemia groups and bore no relation to the use of CEUS. Of note, the four tumours that had PSM is too small of a sample to permit firm interpretation of oncological outcome. In addition, those specimens were among cases of medially located tumour performed during our early learning curve (Figure 1(a), 11 and 12).

Marszalek and colleagues demonstrated PSMs to be present in 0–7% of patients after open PN, 0.7–4% after LPN, and 3.9–5.7% after RPN 28. Similarly, the PSM in this preliminary small series remains comparable and consistent with a recent large series from the Canadian Kidney Cancer Information System Database. This contains rate and predictors of PSM and oncological outcomes in 1066 patients who underwent PN in major academic centers across Canada. PSM occurred in 59 (5.5%) and in multivariate analysis, Fuhrman grade 4 predicted the presence of PSM whereas age, operative technique, tumour size, tumour stage did not. Moreover, PSM after PN did not result in adverse oncological outcomes at mean follow‐up of 18.5 months 29. Similarly, Bensalah *et al*. studied 111 patients with a PSM from a multi‐center survey and compared it with 664 NSM patients. PSM status demonstrated no influence on cancer‐specific survival 30.

In this series, the focus was to elucidate the technical aspects and perioperative outcomes. Our data on long‐term oncological outcomes will be the subject of a larger series and future work.

## Conclusion

A novel technique of real‐time ‘in situ mapping’ and ‘sequential selective occlusion angiography’ for selective ischaemia RPN using intraoperative CEUS is described. Initial experience and results are promising. This technique is safe, feasible and cost‐effective with comparable perioperative outcomes. The technical aspects elucidate the role of intraoperative CEUS and may potentially facilitate and ascertain selective ischaemia during RPN and lower WIT. Further work is required to demonstrate long‐term oncological outcomes.

## Conflict of interest

None
